# *Boswellia serrate* Gum Resin Mitigates Renal Toxicity: Role of TNF-α, Interleukins, TGF-β, and Lipid Peroxidation

**DOI:** 10.3390/life14121669

**Published:** 2024-12-17

**Authors:** Heba M. Eltahir, Abdel-Gawad S. Shalkami, Ahmed M. Shehata, Mohannad Almikhlafi, Ahmed J. Aldhafiri, Ali Alalawi, Muayad Albadrani, Ahmad Bakur Mahmoud, Mekky M. Abouzied

**Affiliations:** 1 Department of Pharmacology and Toxicology (Biochemistry Subdivision), College of Pharmacy, Taibah University, Madinah 41411, Saudi Arabia; htahir@taibahu.edu.sa; 2 Department of Pharmacology and Toxicology, Faculty of Pharmacy, Al-Azhar University, Assiut 71524, Egypt; as.abdulgawad@amc.edu.sa; 3 Clinical Pharmacy Program, College of Health Science and Nursing, Al-Rayan Colleges, Madinah 41411, Saudi Arabia; 4 Pharmaceutical Sciences Department, Fakeeh College for Medical Sciences, Jeddah 21461, Saudi Arabia; amshehata@fcms.edu.sa; 5 Department of Pharmacology and Toxicology, Faculty of Pharmacy, Beni-Suef University, Beni-Suef 62521, Egypt; 6 Department of Pharmacology and Toxicology, College of Pharmacy, Taibah University, Madinah 41411, Saudi Arabia; mmikhlafi@taibahu.edu.sa (M.A.); adhafiri@taibahu.edu.sa (A.J.A.); aalawi@taibahu.edu.sa (A.A.); 7 Department of Family and Community Medicine, College of Medicine, Taibah University, Madinah 41411, Saudi Arabia; mbadrani@taibahu.edu.sa; 8 Health and Life Research Center, Taibah University, Madinah 41411, Saudi Arabia; abamahmoud@taibahu.edu.sa; 9 College of Applied Medical Sciences, Taibah University, Madinah 42353, Saudi Arabia; 10 Department of Biochemistry, Faculty of Pharmacy, Minia University, Minia 61519, Egypt

**Keywords:** renal damage, inflammatory cytokine, TGF-β, *Boswellia serrata*

## Abstract

**Background and aim:** Being a central organ in homeostasis and maintaining the health of the biological system, kidneys are exposed to variable toxicants. Long-term exposure to nephrotoxic molecules causes chronic renal damage that causes fibrosis and loss of function. Such damage can be initiated by oxidative stress which provokes inflammation. We aim at investigating the potential therapeutic effects of Boswellia serrata (BS) gum resin extract in managing CCl_4_-induced renal toxicity. **Methods:** Male Wistar albino rats were assigned to groups: healthy control; CCl_4_-treated (CCl_4_, twice/week, for 6 weeks); CCl_4_ + BS-treated: CCl_4_ for 6 weeks followed by BS (150 mg/kg/day) for 2 weeks; and CCl_4_ + Silymarin-treated: CCl_4_ for 6 weeks followed by Silymarin (100 mg/kg/day) for 2 weeks. Blood and kidney tissue were utilized to assess oxidative stress status, inflammatory cytokines, and histopathological changes. **Results:** BS treatment ameliorated signs of renal damage and fibrosis as it improved renal antioxidant status and renal function markers and significantly reduced the levels of inflammatory cytokines TNF-α, IL-1β, IL-6, and IL-8 along with the fibrogenic marker TGF-β. Kidney tissues showed improved histological features after BS treatment. **Conclusions:** BS gum resin extract has significant therapeutic potential against CCl_4_-induced renal damage and fibrosis. These effects could be mediated via its previously reported antioxidant, free radical scavenging, and anti-inflammatory effects.

## 1. Introduction

Nephrotoxicity is the pathological response of the renal system to various insults such as environmental and biological pollutants and drugs. Exposure of the kidneys to nephrotoxic compounds can induce cellular damage and renal dysfunction [1]. Several molecular mechanisms are activated either to initiate or propagate renal damage such as induction of an inflammatory response, apoptosis, and necrosis, which can all be induced by increasing the oxidative stress within the cell [2]. Prolonged exposure of kidneys to nephrotoxic agents can result in chronic renal damage with the subsequent induction of tubulointerstitial fibrosis and loss of function.

Carbone tetrachloride (CCl_4_) is a clear, lipophilic organic solvent that was formerly used as a detergent, pesticide, refrigerant, and fire extinguisher and was reported to possess acute and chronic hepatic and renal toxicity that may develop into carcinogenesis [3]. CCl_4_ has been widely used to induce experimental hepatic and renal injury and fibrosis in animal models due to its ability to mimic the oxidative stress associated with variable pathological conditions in humans [4]. After administration of CCl_4_, it is metabolized by the microsomal enzyme cytochrome P450 found in the liver and kidney, producing the highly reactive metabolites trichloromethyl (CCl3*) and trichloromethyl peroxyl radicals [3]. These metabolites start attacking polyunsaturated lipids, DNA, and membrane proteins, generating a condition of oxidative stress within the renal and hepatic tissues. Such an oxidative stress condition induces lipid peroxidation and DNA damage, resulting in tissue injury [5]. Furthermore, the produced free radicals deplete the endogenous antioxidants including glutathione, catalase, and superoxide dismutase, which further attenuates the cells’ ability to resist and sequester the ROS-induced damage [6,7]. It has been also reported that CCl_4_ negatively impacts the mitochondrial functions within the renal tissue, affecting calcium movement across the mitochondrial membranes [8]. Overwhelming the antioxidant defense system by exposing it to enormous amounts of ROSs results in pathological responses such as inflammation, adaptation, repair, or injury [9].

Progression of renal injures into fibrosis develops as a reparative response mechanism which is accompanied by loss of the intricate balance between extracellular matrix (ECM) build up and degradation [10].

As mentioned before, oxidative stress induces inflammatory response via inducing the release of various inflammatory cytokines, which play a pivotal role in the initiation and progression of renal inflammation into renal damage.

Tumor necrosis factor-α (TNF-α) as well as interleukins (ILs, e.g., IL-6, IL-8, and IL-1β) represent a large group of pro-inflammatory mediators implicated in many physiological processes such as cellular proliferation and maturation. However, they play a major role in the inflammatory response in biological systems. Based on their latter-mentioned role, some reports demonstrated promising results for targeting and inhibiting these mediators in ameliorating nephrotoxicity among other inflammatory diseases [11,12].

Transforming growth factor-β (TGF-β) is another important pro-fibrotic mediator that is involved in ECM deposition and hence the development of renal fibrosis [13].

Several reports have proven the efficacy of using natural drugs known for their antioxidant properties in combating oxidative stress and, hence, alleviating the subsequent cellular events. These compounds exert their effects by replenishing the endogenous antioxidant capacity, scavenging free radicals, and/or affecting the metabolizing enzymes so they stop producing more ROSs [14,15].

Gum resin extracted from Boswellia serrata (BS) is commonly used in traditional herbal medicine as it possesses multiple therapeutic effects such as anti-inflammatory, antirheumatic, antihyperlipidemic, and hepatoprotective effects [16,17,18]. BS has many active constituents including boswellic acids, Acetyl-β-Boswellic acid, 11-keto-β-Boswellic acid, and 3-acetyl-11-ketobeta-boswellic acid (AKBA) [17,19].

BS gum resin was shown to ameliorate inflammation by inhibiting the expression of many inflammatory cytokines such as TNF-α, IL-1, IL-2, and IL-6 as well as inhibiting the activity of 5-lipooxygenase which all play a crucial role in the inflammatory response [20,21,22]. In addition, it was reported to alleviate CCl_4_-induced liver fibrosis by modifying the expression of the pro-fibrotic mediator TGF-β [18]. Renal fibrosis represents a major challenge as it takes place in response to chronic inflammation and significantly affects renal function. This increases the need for new antifibrotic treatment strategies that would possibly save patients from encountering end-stage renal failure consequences. In the current study, we focused on investigating the effects of a BS extract in ameliorating CCl_4_-induced nephrotoxicity

## 2. Materials and Methods

**Materials**

Chemicals used in the current study were purchased from Sigma-Aldrich and were all of analytical grade. The *Boswellia serrata* (BS) oleo-gum resin used in this study was a kind gift from Prof. Dr H. Ammon, Department of Pharmacology, Institute of Pharmaceutical Sciences, University of Tuebingen, Germany (Tubingen, Germany).

As previously published, BS oleo-gum resin is composed of 3-O-acetyl-11-keto-β-boswellic acid (AKBA), 3-O-acetyl-α-boswellic acid, and 11-keto-β-boswellic acid (KBA, derivatives of α and β boswellic acids) [18,23]. Using a mobile phase composed of acetonitrile/water/ortho-phosphoric acid 85% (50:40:9.5:0.5 *v*/*v*), HPLC was utilized to analyze the oleo-gum resin extract according to the method of Tawab et al. [24] with slight modification (Shimadzu LC-10AT pump, SIL-10AD autosampler, SCL-10A controller and SPD-10A detector, flow rate 1.0 mL/min, and UV detection at 250 nm). For the analysis, a LiChrospher 100, RP-18 Merck column, 5 µm (125 × 4 mm) was used with the corresponding LiChrospher 100, RP-18 Merck column, 5 µm (4 × 4 mm) guard columns. To generate standard curves, the corresponding peak was plotted against a standard concentration of KBA or AKBA. These curves were then used to calculate the amount of KBA and AKBA in the gum resin.

**Animal experiment**

Thirty-two adult male Wistar albino rats weighing 200–250 g were used in this study. Rats were obtained from the animal house, faculty of medicine, Assiut University. All animals received suitable care at all times during the experiments and had free access to water and food. The animal experimental procedures were approved by the research ethics committee, Faculty of Pharmacy, Al-Minia University, Egypt. All experimental procedures including live animals were performed according to the “international ethical guidelines for animal care of the United States Naval Medical Research Centre”, Unit no. 3, Abbaseya, Cairo, Egypt, accredited by the “Association for Assessment and Accreditation of Laboratory Animal Care International”.

Animals were allowed to accommodate for one week before starting the experimental procedures and being divided into four groups (8 rats per group).

**Control group**: received 1 mL/Kg of olive oil, intra-peritoneal (I.P.), twice weekly for 6 weeks;

**CCl_4_-treated group**: renal toxicity was induced in this group by injection of CCl_4_ (1 mL/kg of 40% CCl_4_ in olive oil *v*/*v*), I.P., twice weekly for 6 weeks [25];

**BS-treated group**: renal toxicity was first induced in rats by injection of CCl_4_ for 6 weeks like group B, and then, BS treatment was initiated as they were given 150 mg/kg/day I.P. of BS for two weeks starting after the end of CCl_4_ treatment [26];

**SIL-treated group**: renal toxicity was induced by injection of CCl_4_ like group B. Then, Silymarin treatment was initiated as rats were given Silymarin (100 mg/kg/day, orally) for two weeks starting after the end of CCl_4_ treatment [27].

By the end of the experiment (end of week 8), rats were anesthetized by ketamine (100 mg/kg) and xylazine (20 mg/kg), and blood samples were collected using cardiac puncture. Animals were then sacrificed under deep anesthesia and kidneys were collected for further examinations.

Blood samples were centrifuged at (3000 rpm) for 20 min to separate serum; then, sera were stored at −20 until being used for biochemical analysis. Kidneys were excised promptly after sacrifice, rinsed in ice-cold normal saline, and divided into two segments; one was fixed in buffered formalin solution for histological investigation and the second was homogenized in ice-cold homogenization buffer then centrifuged for 10 min (3000 rpm), where the supernatant was used for the different biochemical analysis.

**Determination of renal function biomarkers**

Serum levels of creatinine and urea were evaluated for assessing renal function. Serum urea and creatinine levels were measured using commercially available kits (Bio-Diagnostics, Egypt) following the manufacturer’s instructions.

**Assessment of oxidative stress biomarkers**

Kidney homogenates were used to measure the content of malondialdehyde (MDA) as a marker of lipid peroxidation. MDA content was determined calorimetrically depending on the reaction of MDA with thiobarbituric acid [28]. Catalase activity was determined using the method of Claiborne [29]. In addition, the total antioxidant capacity was evaluated using commercially available kits based on the previously published method [30] (CUSABIO, USA).

**Estimation of TNF-α and TGF-β1 levels in renal tissue**

Determination of tissue contents of TNF-α and TGF-β1 in kidney homogenates was performed using commercially available ELISA kits according to the manufacturer’s instructions (CUSABIO, USA).

**Estimation of IL-1β, IL-6 and IL-8**

Serum levels of some interleukins including IL-1β, IL-6, and IL-8 were assessed using commercially available enzyme-linked immune-sorbent assay kits (ELISA) following the manufacturer’s instructions (Aviva Systems Biology, San Diego, CA, USA).

**Histopathological analysis**

For the histopathological investigation, fixed kidney tissues were dehydrated in serial dilutions of ethanol and embedded in paraffin. Then, 5 μ-thick sections were cut and stained with hematoxylin and eosin (H&E), Masson’s Trichrome, and Periodic Acid Schiff’s stain (PAS). Stained sections were examined under a light microscope (Zeiss Axiovert A1.0) and evaluated by a specialized histopathologist.

**Statistical analysis**

Obtained data were analyzed using GraphPad Prism 6 and are presented as mean ± SEM. The Student’s *t*-test was used to evaluate significance, where *p* values less than 0.05 indicated a significant difference. Statistical analysis was conducted by one-way ANOVA followed by the Tukey–Kramer post hoc test for multiple comparisons between groups.

## 3. Results

### 3.1. HPLC Analysis and Characterization of BS Oleo-Gum Resin

HPLC analysis showed that KBA and AKBA had a retention time of 4.5 and 10.4 min, respectively, which were comparable to the retention times of the standard compounds. The concentrations of both acids in the BS gum resin were 5.48% and 4.66%, respectively, based on the UV absorption [18,23].

### 3.2. Effect of BS on Renal Function Parameters

Administration of CCl_4_ resulted in a significant three-fold elevation in serum creatinine and two-fold increase in urea levels compared to the healthy control group (*p* < 0.05). Interestingly, treatment with either BS or SIL significantly ameliorated the increase in serum levels of creatinine and urea with respect to CCl_4_-treated animals that did not receive further treatment (Table 1). It is to be noted that BS treatment resulted in serum urea and creatinine values that were not distinguishable from healthy control values (Table 1).

### 3.3. Effect of BS Treatment on Oxidative Stress Markers

A significant increase in renal MDA content was observed in the rats that received CCl_4_ compared to healthy control rats. Treatment of rats with BS caused a significant decrease in renal tissue content of MDA compared to CCl_4_-treated animals (*p* < 0.05). On the other hand, CCl_4_ treatment resulted in a significant reduction in catalase activity compared to healthy control animals. Notably, BS treatment significantly retrieved catalase activity in renal tissue compared to CCl_4_-treated animals as presented in Table 1 (*p* < 0.05).

Additionally, a significant depletion in total antioxidant capacity was observed in CCl_4_-treated rats compared to healthy control animals. Treatment with BS after induction of renal injury with CCl_4_ significantly retrieved the total antioxidant capacity as compared with the CCl_4_-treated group.

Similarly, administration of SIL resulted in a significant reduction in MDA content and retrieval of CAT activity and total antioxidant capacity in comparison to the CCl_4_-treated group (Table 1).

### 3.4. Effect of BS Treatment on Inflammatory Cytokine Levels After Renal Damage Induction

**Figure 1:**
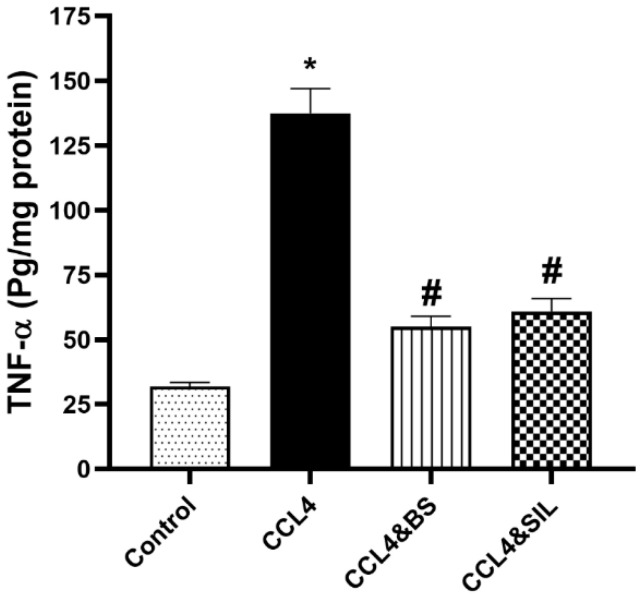
**Effect of the different treatments on TNF-α level in CCl_4_-induced renal toxicity.** CCl_4_ administration resulted in a significant increase in TNF-α levels compared to healthy control animals. Treatment of animals with BS or SIL after the establishment of renal damage significantly ameliorated TNF-α levels compared to animals receiving CCl_4_ without further treatment. Data are represented as mean ± SEM. *: values are significantly different from healthy control; #: values are significantly different from CCl_4_-treated animals. *p* < 0.05.

As compared with the healthy control group, administration of CCl_4_ induced a significant increase in the level of TNF-α. BS treatment of renal-damaged animals resulted in a significant reduction in TNF-α levels compared to CCl_4_-treated animals. In a similar attitude, administration of SIL also resulted in a significant reduction in the TNF-α level as compared with the model group (Figure 1, *p* < 0.05).

**Figure 2:**
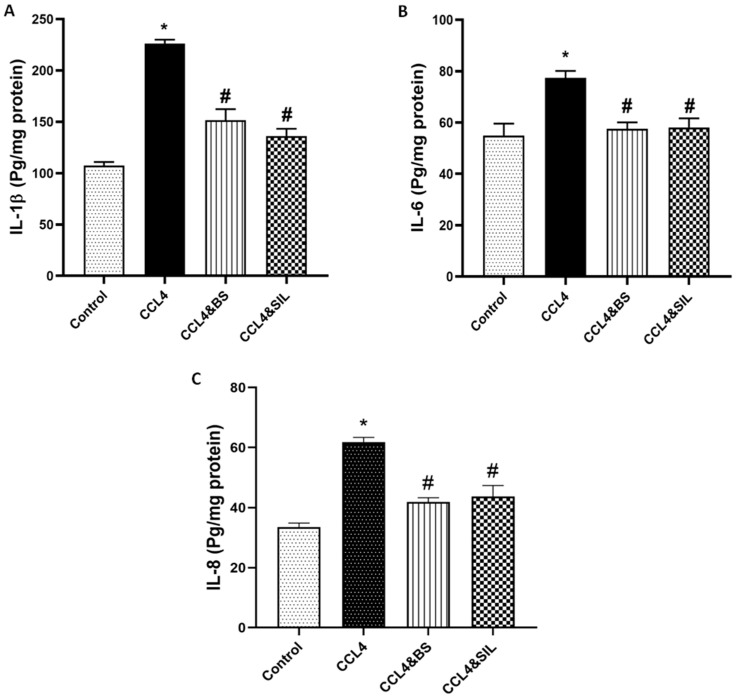
**Effect of the different treatments on IL-1β, IL-6, and IL-8 levels in CCl_4_-induced renal toxicity.** CCl_4_ administration resulted in a significant increase in the levels of all three interleukins IL-1β (**A**), IL-6 (**B**), and IL-8 (**C**) compared to healthy control animals. Treatment of animals with BS or SIL after establishment of renal damage significantly ameliorated the levels of these interleukins compared to animals receiving CCl_4_ without further treatment. Data are represented as mean ± SEM. *: values are significantly different from healthy control; #: values are significantly different from CCl_4_-treated animals. *p* < 0.05.

By investigating the level of other inflammatory cytokines, IL-1β, IL-6, and IL-8, one can observe a significant increase in their levels upon treatment with CCl_4_ compared to healthy control animals (*p* < 0.05). In contrast, BS as well as SIL administration significantly ameliorated the levels of these cytokines compared to animals that received CCl_4_ only (*p* < 0.05, Figure 2A–C).

### 3.5. Effect of BS on TGF-β Levels After Renal Damage Induction

**Figure 3:**
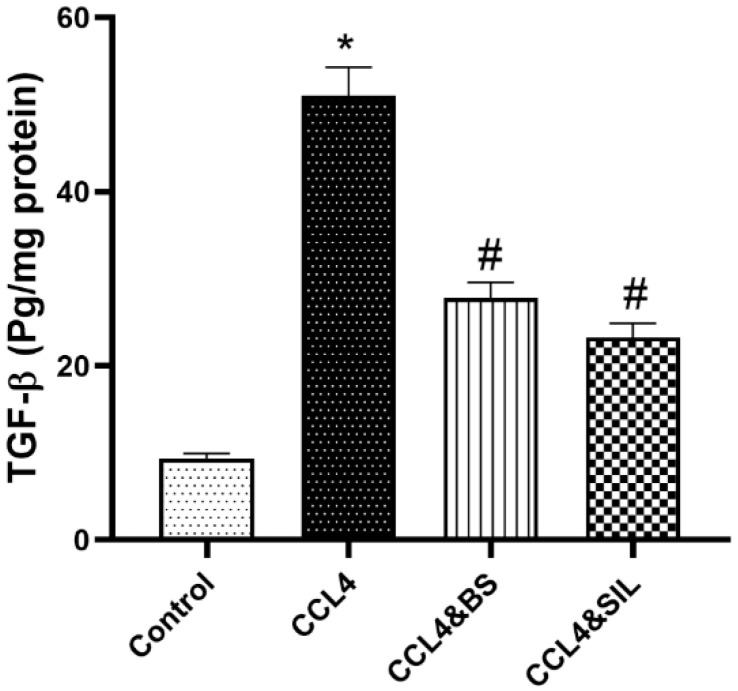
**Effect of the different treatments on TGF-β levels in CCl_4_-induced renal toxicity.** CCl_4_ administration resulted in a significant increase in TGF-β levels compared to healthy control animals. Treatment of animals with BS or SIL after the establishment of renal damage significantly ameliorated TGF-β levels compared to animals receiving CCl_4_ without further treatment. Data are represented as mean ± SEM. *: values are significantly different from healthy control; #: values are significantly different from CCl_4_-treated animals. *p* < 0.05.

Administration of CCl_4_ induced a significant increase in TGF-β levels in comparison to healthy control animals (*p* < 0.05). Interestingly, administration of BS or SIL to CCl_4_-intoxicated animals was able to cause a significant reduction in the level of TGF-β compared to CCl_4_-treated animals that did not receive further treatment (*p* < 0.05, Figure 3).

### 3.6. Effect of BS on Kidney Histology After Induction of Renal Damage

**Figure 4:**
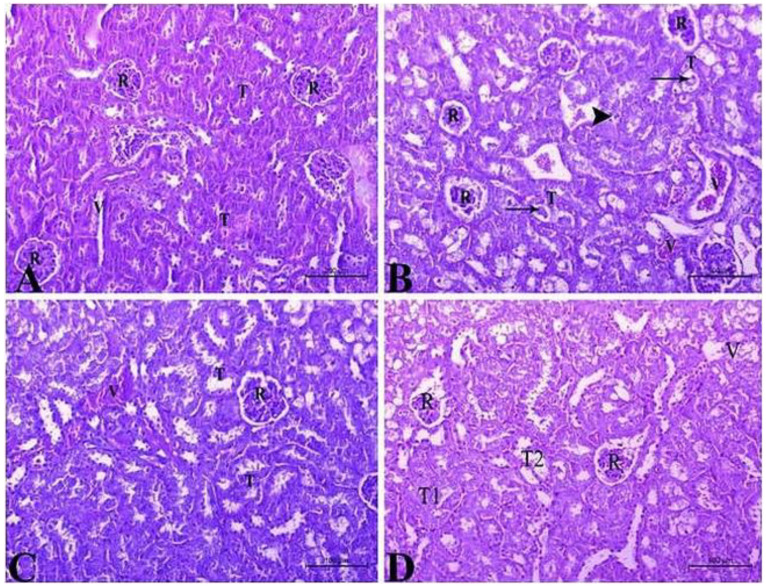
**Effect of the different treatments on renal histological architecture.** (**A**) Photomicrograph of kidney section from control animals showing normal renal architecture including normal renal corpuscles (R) and renal tubules (T) as well as intact, healthy blood vessels (V). (**B**) sections from renal tissue of CCl_4_-treated animals showing atrophy of the renal corpuscle (R) and severe degenerative changes in the renal tubules epithelium (T), as well as cystic dilatation and hyaline cast appearance (arrow). Infiltrating lymphocytes are visible in the interstitial tissue (arrowhead), and renal blood vessels (V) appear highly congested. (**C**) Renal tissue sections from CCl_4_-treated rats administered BS showing almost normal renal corpuscles (R) and renal tubules (T). Renal blood vessels (V) show signs of mild congestion. (**D**) Renal tissue sections from CCl_4_-treated rats administered Silymarin showing normal renal corpuscles (R) and renal tubules (T1); however, some tubules (T2) showed degenerative changes in their epithelia. Also, mildly congested renal blood vessels (V) could be observed. Hematoxylin and eosin staining; bars = 100 µm; 200×.

Kidney sections from healthy control rats showed normal histological features of renal tubules and glomeruli with no signs of congestion or inflammatory cell infiltration as observed in sections stained with hematoxylin and eosin (Figure 4A). By the end of the CCl_4_ treatment course, kidney sections revealed pronounced histopathological changes, such as perivascular edema and inflammatory cell infiltration. In Figure 4B, cystic dilatation, epithelial damage of tubules, and congestion of the glomerular tuft and renal blood vessels can be observed. Sections from rats which received BS or SIL after CCl_4_-intoxication reflected relative histological improvement, showing almost normal corpuscles and tubules with mildly congested blood vessels (Figure 4C,D).

**Figure 5:**
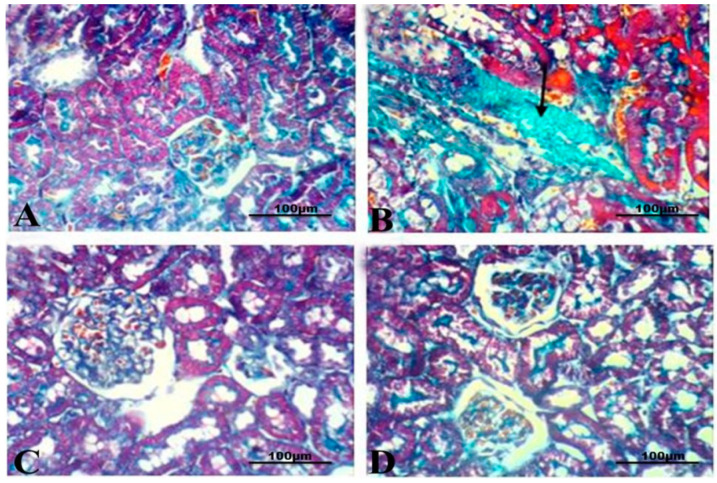
**Effect of the different treatments on ECM and collagen deposition.** (**A**) Photomicrograph of kidney section from control animals stained with Masson’s Trichrome stain showing no accumulation of collagen fibers. (**B**) Kidney sections from CCl_4_-treated group showing a strong positive staining for densely accumulated collagen fibers (arrows). (**C**,**D**) Kidney sections from BS- and SIL-treated animals, respectively, showing almost no accumulation of collagen fibers (Masson’s Trichrome stain, bars = 50 µm, and 400×).

In order to investigate the changes in the extracellular matrix, sections were stained with Masson’s Trichrome staining. Kidney sections from the healthy control group exhibited no signs of collagen accumulation, indicating no fibrotic changes within the renal tissue (Figure 5A). In contrast, sections from kidneys of CCl_4_-treated rats displayed a prominent accumulation of dense collagen fibers indicating ECM deposition and fibrosis, in addition to dilated and atrophied tubules (Figure 5B). Notably, kidney sections from rats treated with BS or SIL after renal damage showed minimal deposition of ECM around the blood vessels compared to CCl_4_-treated animals (Figure 5C,D).

**Figure 6:**
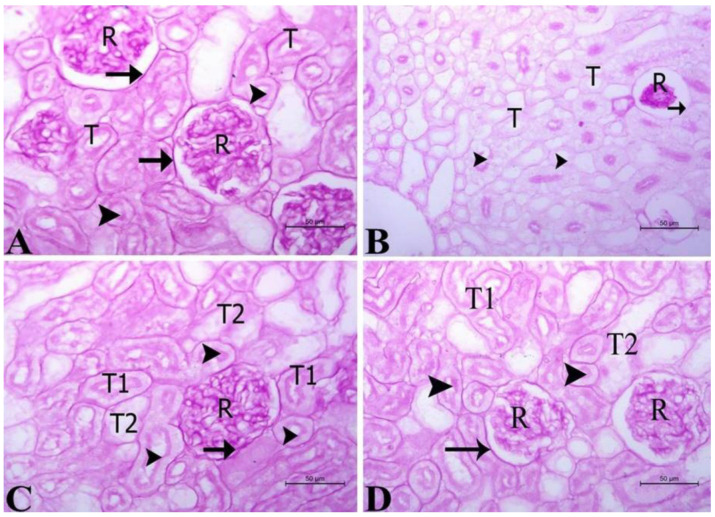
**Effect of the different treatments on basement membrane integrity.** (**A**) A kidney section from the control group showing normal renal corpuscles (R) enclosed by intact basal lamina (arrow) and renal tubules (T) lined with tubular epithelium with normal apical brush borders (arrow head). (**B**) A kidney section from the CCl_4_-treated group showed partially lost brush borders (arrowhead), dilated renal tubules (T), and deformed basal lamina surrounding the renal corpuscle (arrow). (**C**) A kidney section from CCl_4_-treated rats administered BS showing intact basal lamina enclosing the renal corpuscle (R) (arrow); brush borders are partially lost in few renal tubules (T2), while the majority of tubules (T1) appeared normal with an apical brush border (arrow head). (**D**) A kidney section from CCl_4_-treated rats administered Silymarin showing a renal corpuscle (R) enclosed by a thick basal lamina (arrow) as well as a lost brush border and cystic dilatation in some renal tubules (T2), while the other tubules (T1) appeared normal with an apical brush border (arrow head). PAS staining; bars = 50 µm.

For assessment of the integrity of the basement membranes in corpuscles and vessels, kidney sections from the different test groups were stained with Periodic Acid Schiff’s stain (PAS, Figure 6). Healthy kidney sections showed intact corpuscles with normal basal lamina as well as normal brush borders within the renal tubules (Figure 6A). After induction of renal damage with CCl_4_, brush borders were partially lost and cystic dilatation along with distorted basal lamina were detected (Figure 6B). Interestingly, treating animals with BS after CCl_4_-induced renal damage successfully alleviated histopathological changes, where renal corpuscles appeared normal with intact lamina. Most of the renal tubules regained intact brush borders as seen in Figure 6C. It is to be noted that treating animals with the standard drug Silymarin also resulted in improved tissue integrity with few damaged brush boarders and dilated tubules (Figure 6D).

## 4. Discussion

The kidney represents a vital organ that plays crucial roles to keep a balanced and healthy biological system. It is responsible for maintaining homeostasis and acid–base balance, as well as regulating blood pressure and filtering the blood from the un-needed and harmful molecules. This last function exposes the kidneys to a wide range of hazardous compounds and increases its vulnerability to such insults [31]. Excessive exposure of the renal tissue to these hazardous molecules such as cisplatin, diethylnitrosamine, amikacin, and CCl_4_ induces a condition of nephrotoxicity, which is characterized by a decline in renal functions that may develop into renal dysfunction [1].

In the current study, we aimed at investigating the therapeutic effect of BS in ameliorating CCl_4_-induced renal damage. Several experimental animal models have been utilized to investigate the pathology of renal damage and the potential therapeutic effect of a wide range of promising drugs. CCl_4_-induced damage is one of the commonly used experimental models for renal and hepatic damage as well. The effect of CCl_4_ relies on the initiation of oxidative stress as a result of its metabolism into a group of highly reactive metabolites [32].

CCl_4_ is an organic solvent that was formerly used for household and industrial cleaning purposes and as a chemical intermediate. Several reports have linked exposure to CCl_4_ with acute and chronic damage to different body organs including the liver, kidney, lungs, and brain. This solvent is metabolized by the hepatic and renal cytochrome P450 enzyme system resulting in the production of the highly reactive metabolites trichloromethyl radicals (•CCl3) and trichloromethyl peroxyl radicals (•OOCCl3) that are both classified as reactive oxygen species (ROSs) [33,34]. Being very reactive, these ROSs attack and destruct many cellular components including bio-membranes, proteins, and DNA with subsequent cell dysfunction. It is to be mentioned that the kidney also contains cytochrome P450, which converts CCl_4_ to its toxic metabolites locally in the cortical cells of renal tubules [35].

Excessive production of ROSs as a result of CCl_4_ metabolism within the renal tissue initiates a condition of oxidative stress due to the direct damaging effect of the free radicals and their antioxidant-depleting effects as well, resulting in lipid peroxidation, DNA strand breaks, and damage to bio-membranes [36]. Oxidative stress also induces the inflammatory response which is linked to a wide range of pathological changes that may end up with organ damage [37].

The reactive metabolites of CCl_4_ initiate a chain of lipid peroxidation which can drastically alter the characteristics of biological membranes, leading to severe cellular injury and playing an important role in nephrotoxicity [38]. As observed in the current study, administration of CCl_4_ induced a significant elevation in renal MDA, indicating lipid peroxidation, and a significant increase in serum urea and creatinine levels, which signifies the negatively impacted renal functions due to deterioration of the structural integrity of the kidney functional units.

Catalase is an endogenous antioxidant enzyme that is responsible for converting reactive hydrogen peroxide (H_2_O_2_) into harmless water [39]. Similarly, thiols which include glutathione and thiol-containing proteins are considered the largest constituent of the endogenous antioxidant defense mechanisms [40]. In agreement with previously published reports, the current study shows a significant decrease in endogenous antioxidants after administration of CCl_4_. This can be attributed to the oxidative stress induced by CCl_4_ metabolites which overwhelms and depletes the endogenous antioxidants, as well as renal lipid peroxidation, which was shown by others to cause defects in endogenous antioxidant defense mechanisms [41,42,43].

It is to be noted that renal dysfunction resulting from a chronic inflammatory response represents an economic and health burden as it is usually associated with a high rate of mortality and morbidities, e.g., hypertension, increased cholesterol levels, and diabetes [44]. The relation between oxidative stress and inflammation is a complex, mutual relationship, where oxidative stress promotes the inflammatory response, and vice versa, the inflammation aggravates the oxidative stress state at the inflammation site [45,46]. In response to oxidative stress and the damage on the cellular and molecular levels, macrophages and renal tubular cells start to release a group of pro-inflammatory mediators such as ILs and TNF-α, which aid and promote the inflammatory response [47,48].

In the current work, CCl_4_ treatment depleted the endogenous antioxidant capacity represented by the decreased total thiol content and induced a sterile inflammatory response as observed by the increased levels of the proinflammatory cytokines IL-1β, IL-6, IL-8, and TNF-α. These findings are similar to the results reported by others [49,50]. In addition to its physiological roles in cell growth, division, and apoptosis, TNF-α is a key inflammatory mediator that plays a role in the activation and nuclear translocation of nuclear factor κB (NF-κB) and, hence, the transcriptional activation of NF-κB downstream pro-inflammatory cytokines and interleukins [49]. Within the renal system, TNF-α is expressed by renal tubular cells and mesangial cells, where some reports have linked the increase in its levels to nephrotoxicity and renal complications [51]. Other studies showed the beneficial effect of inhibiting TNF-α expression on preventing the infiltration of inflammatory cells and ameliorating fibrosis [52]. Interleukins represent a group of cytokines that are produced and released by various cell types including immune cells as well as local tissue cells. Different ILs such as IL-1, IL-6, and IL-8 have been considered as proinflammatory mediators in chronic renal dysfunction [51,53].

Due to the oxidative stress status initiated by the action of CCl_4_ and its metabolites, inflammasomes are activated. These are intracellular proteins that drive the inflammatory response by cleaving pro-caspase-1 into active caspase-1, which in turn forces the maturation and release of other cytokines, specifically IL-1β [54]. IL-1β acts as a pro-inflammatory mediator that activates macrophages, inducing them to release further inflammatory cytokines and also activating cyclooxygenase-2 to produce PGE2, which plays a main role in renal damage [55,56]. CCl_4_ administration was shown in the literature to induce the expression of IL-1β and IL-6 as well as TNF-α [43,57]. In addition, IL-1β was shown to mediate fibrocyte differentiation and interstitial fibrosis, adding to its central role in renal pathogenesis [58,59]. Some studies reported that interfering with IL-1β and with the inflammasome-driven IL-1β release can be of value in variable inflammatory disorders including chronic nephropathy [60].

Both TNF-α and IL-1β function to stimulate the production and release of IL-6 from immune cells among others [61]. IL-6 is a pleiotropic cytokine that regulates the inflammation process and immune responses in addition to its central role in multiple physiological and developmental processes. Within the renal tissue, IL-6 is secreted by several cell types including mesangial and tubular endothelial cells where it activates immune and inflammatory cells, mediating renal injury. In addition, IL-6 promotes mesangial proliferation and ECM accumulation [62].

IL-8 is a chemotactic mediator that is secreted by different cell types including macrophages, where it attracts neutrophils and other inflammatory cells to the inflammation site, modulating cell growth and aiding in angiogenesis [63,64]. Previous studies reported an increase in IL-8 levels in animal models of CCl_4_-induced renal damage [65]. Elevated IL-8 levels augment the immune response to the inflammatory signals, resulting in the subsequent release of other inflammatory cytokines from the infiltrated immune cells and progression of the inflammation and tissue injury [66].

As a consequence of progressive chronic renal injury and inflammation in response to CCl_4_ exposure or any other etiology, renal fibrosis with tissue scarring, loss of structure integrity, and end-stage renal disease could be induced [6]. Renal fibrosis is characterized by excessive deposition of collagen and other extracellular matrix (ECM) components, which is mediated by active myofibroblasts [67]. It is to be noted that the inflammatory response and renal injury provoke the damaged tubular endothelial cells and the infiltrating immune cells to release variable signals, which consequently activate the myofibroblasts, causing them to increase ECM deposition [68]. The increased collagen synthesis in mesangial cells was shown to increase vascular mineralization and negatively impact renal function [69]. TGF-β is a multifunctional cytokine responsible for the regulation of different physiological functions such as cell proliferation, differentiation, and apoptosis. On the other hand, its expression was also shown to be upregulated in response to oxidative stress and inflammation as observed in injured human and animal kidneys [13,70]. In such pathological conditions, it plays a central role as a fibrogenic factor by increasing the synthesis of ECM components including collagens and fibronectin and interfering with the production of metalloproteinases that are responsible for ECM degradation [70,71]. Some studies reported a role for TGF-β in activating COX-2 and, hence, increasing PGE2 levels, which was linked to the progression of chronic kidney disease [72]. It is to be mentioned that TGF-β is a central player in initiating and maintaining renal fibrosis via multiple signaling pathways including Smad signaling, where neutralization of TGF-β on the molecular level effectively attenuated renal fibrosis [70]. The present study showed an increase in the level of TGF-β in CCl4-treated rats compared to the control group which supports the role of TGF-β in the induction of renal fibrosis. In the current study, we observed an increase in fibrosis markers both on the histological as well as the molecular level, where both ECM deposition and TGF-β levels were increased in animals treated with CCl_4_, confirming the previously reported fibrogenic effect of CCl_4_ [18,73].

A large number of studies revealed the efficacy of natural compounds in alleviating and managing renal toxicity and renal damage based on their antioxidant, anti-inflammatory, and antifibrogenic properties [74,75,76].

Boswellia serrata (BS) extract was used for the management of different inflammatory diseases in experimental models of rheumatoid arthritis, ulcerative colitis, and liver fibrosis [13,77]. In the current study, we observed an ameliorative effect for BS against CCl_4_-induced renal damage in the form of improved renal functions (measured by blood urea nitrogen and serum creatinine levels) and retrieved endogenous antioxidant capacity. In addition, BS treatment normalized the oxidative stress status, reduced inflammation, and mitigated fibrosis as observed by the significant reduction in levels of TNF-α, IL-1β, IL-6, IL-8, and TGF-α in the treatment group compared to the animals receiving CCl_4_ alone. This therapeutic effect of BS was confirmed on the histological level too.

These promising effects for BS can be attributed mainly to its previously reported antioxidant and anti-inflammatory properties and its ability to reduce the expression of pro-inflammatory cytokines [18,77,78]. It is to be noted that the antifibrotic effect of BS observed in the current study was reported in previous studies investigating its effect in an experimental model for liver fibrosis [18].

Gum resin of BS contains tetracyclic and pentacyclic phenolic compounds [79]. These phenolic compounds allow the BS extract to scavenge free radicals and support the endogenous antioxidant mechanisms. In addition, some studies reported the value of using antioxidants and ROSs scavenging molecules in cytochrome P450-mediated oxidative stress, where these molecules interfere with the enzyme’s ability to metabolize xenobiotics into toxic, reactive metabolites.

## 5. Conclusions

In conclusion, the current study elucidates the therapeutic effect of BS against CCl_4_-induced nephrotoxicity and fibrosis. The observed positive effects of BS can be attributed mainly to its antioxidant, free radical scavenging, and anti-inflammatory properties. These effects were detectable on the molecular as well as the histological level, as it ameliorated the levels of TNF-α, IL-1β, IL-6, IL-8, and TGF-β as well. Based on the data presented in this work, administration of BS is a promising potential therapeutic natural drug that can mitigate the renal damage induced by CCl_4_ administration.

### Study Limitation

In the current study, we used Silymarin as a standard nephroprotective agent; however, the controversy about its efficacy raises a limitation to the current work. It would be of great value if the nephroprotective effect of Silymarin can be compared to other standard, well-known agents such as betaine, dibenzazepine, or linalool to justify its effects in the current context.

## Figures and Tables

**Table 1:** Effect of BS on renal function and oxidative stress biomarkers in CCl_4_-induced renal toxicity.

	Control	CCl_4_	CCl_4_ + BS	CCl_4_ + SIL
Creatinine (mg/dL)	0.66 ± 0.05	2.05 ± 0.13 *	0.67 ± 0.06 #	0.73 ± 0.04 #
Urea (mg/dL)	41.67 ± 2.62	91.18 ± 7.70 *	53.50 ± 3.14 #	50.41 ± 3.60 #
MDA (nmol/g tissue)	3.44 ± 0.21	7.21 ± 0.50 *	4.80 ± 0.25 #	5.11 ± 0.43 #
CAT (U/mg protein)	168.11 ± 7.88	56.93 ± 3.93 *	91.71 ± 5.32 #	99.13 ± 5.56 #
Total Antioxidant Capacity (U/mg protein)	1.23 ± 0.06	0.40 ± 0.03 *	0.76 ± 0.05 #	0.77 ± 0.04 #

Data are represented as mean ± SEM. *: indicates significant difference compared to healthy control; #: indicates significant difference compared to CCl_4_-treated animals. Significance is considered at *p* values < 0.05. Statistical analysis was conducted by one-way ANOVA followed by a Tukey–Kramer post hoc test for multiple comparisons between groups.

## Data Availability

The original contributions presented in this study are included in this article, further inquiries can be directed to the corresponding author.
